# The prognostic effect of DDX3 upregulation in distant breast cancer metastases

**DOI:** 10.1007/s10585-016-9832-8

**Published:** 2016-12-20

**Authors:** Marise R. Heerma van Voss, Willemijne A. M. E. Schrijver, Natalie D. ter Hoeve, Laurien D. Hoefnagel, Quirine F. Manson, Elsken van der Wall, Venu Raman, Paul J. van Diest, Dutch Distant Breast Cancer Metastases Consortium

**Affiliations:** 1 Department of Pathology, University Medical Center Utrecht, Utrecht, The Netherlands; 2 Department of Radiology and Radiological Sciences, School of Medicine, Johns Hopkins University, Baltimore, MD USA; 3 Cancer Center, University Medical Center Utrecht, Utrecht, The Netherlands; 4 Department of Oncology, School of Medicine, Johns Hopkins University, Baltimore, MD USA

**Keywords:** DEAD box RNA helicases, DDX3, DDX3X, Breast cancer, Metastasis, Brain metastasis

## Abstract

Metastatic breast cancer remains one of the leading causes of death in women and identification of novel treatment targets is therefore warranted. Functional studies showed that the RNA helicase DDX3 promotes metastasis, but DDX3 expression was never studied in patient samples of metastatic cancer. In order to validate previous functional studies and to evaluate DDX3 as a potential therapeutic target, we investigated DDX3 expression in paired samples of primary and metastatic breast cancer. Samples from 79 breast cancer patients with distant metastases at various anatomical sites were immunohistochemically stained for DDX3. Both cytoplasmic and nuclear DDX3 expression were compared between primary and metastatic tumors. In addition, the correlation between DDX3 expression and overall survival was assessed. Upregulation of cytoplasmic (28%; OR 3.7; p = 0.002) was common in breast cancer metastases, especially in triple negative (TN) and high grade cases. High cytoplasmic DDX3 levels were most frequent in brain lesions (65%) and significantly correlated with high mitotic activity and triple negative subtype. In addition, worse overall survival was observed for patients with high DDX3 expression in the metastasis (HR 1.79, p = 0.039). Overall, we conclude that DDX3 expression is upregulated in distant breast cancer metastases, especially in the brain and in TN cases. In addition, high metastatic DDX3 expression correlates with worse survival, implying that DDX3 is a potential therapeutic target in metastatic breast cancer, in particular in the clinically important group of TN patients.

**Electronic supplementary material:**

The online version of this article (doi:10.1007/s10585-016-9832-8) contains supplementary material, which is available to authorized users.

## Background

DDX3 (encoded by *DDX3X*) is a DEAD box RNA helicase with ATPase dependent helicase activity, which is involved in several steps of endogenous RNA metabolism and translation initiation [1–4]. DDX3 has been implicated in neoplastic transformation due to its role in cell cycle progression [5, 6] and its anti-apoptotic properties [7–9]. In addition, DDX3 has been shown to promote several steps of tumor metastasis. Overexpression of DDX3 resulted in increased motility and migration by induction of an epithelial-to-mesenchymal (EMT) phenotype with loss of E-cadherin [10, 11] and upregulation of Snail expression [12]. Furthermore, DDX3 was found to promote anchorage independent growth and invasive capacities of cancer cells through regulation of mRNA translation [13, 14]. DDX3 knockdown has also been shown to result in reduced breast cancer metastases in mice [15]. These findings have led to the development of DDX3 inhibitors for the treatment of breast cancer [15] among other malignancies [5, 6, 16, 17]. The tumor-enhancing role of DDX3 was corroborated by studies on DDX3 expression in patient samples of primary tumors [5, 6], but DDX3 expression was never studied in metastatic cancer samples.

Although therapeutic options for patients with metastatic breast cancer have increased, the vast majority of patients still develops resistance to treatment and eventually succumbs to the disease [18]. With 5-year survival rates of 25% [19] and approximately 40,000 deaths on a yearly basis in the United States, metastatic breast cancer still ranks second on the list of causes of cancer deaths in women, accounting for 15% of all cancer deaths [20]. Therefore the identification of novel therapeutic targets that inhibit the development and outgrowth of breast cancer metastases remains urgently wanted.

Upregulation of DDX3 in metastases would confirm the role of DDX3 in metastatic tumor progression that has been suggested in functional studies. In addition, high DDX3 expression levels in metastatic lesions could indicate that breast cancer metastases are reliant on high DDX3 expression, and that patients with advanced disease could benefit from treatment with DDX3 inhibitors under development. Therefore, this study aimed to evaluate DDX3 expression in distant breast cancer metastases as compared to their primary tumor.

## Methods

### Patient samples

Tissue microarrays (TMAs) containing paired samples from 97 primary breast cancer and their distant metastases were previously assembled [21, 22]. All TMAs included multiple cores per patient. 18 pairs were incomplete due to damaged or detached cores during cutting or staining, or due to cores no longer containing invasive carcinoma. The TMA included metastases from various anatomical sites, including brain, skin, lung, liver, bone, ovaries, uterus and the gastro-intestinal tract. Clinicopathological data and follow up data were retrieved from the pathology reports and patient files. Overall survival was calculated from the time of diagnosis of the metastatic lesion. For this study only anonymous archival leftover pathology material was used. Therefore no informed consent is required according to Dutch legislation [23], as this use of redundant tissue for research purposes is part of the standard treatment agreement with patients in the UMC Utrecht [24].

### Immunohistochemistry

Four µm thick sections were cut, mounted on Surgipathe X-tra adhesive slides (Leica Biosystems, Milton Keynes, UK), deparaffinized in xylene and rehydrated in decreasing ethanol dilutions. Endogenous peroxidase activity was blocked with 1.5% hydrogen peroxide buffer for 15 min and was followed by antigen retrieval by boiling for 20 min in EDTA buffer (pH 9.0). Slides were blocked with protein block from Novolink Polymer Detection System (Leica Microsystems, Eindhoven, The Netherlands) and subsequently incubated in a humidified chamber for 1 h with anti-DDX3 (1:50, mAb AO196) [25]. Post primary block, secondary antibodies and diaminobenzidine treatment were performed with the same Novolink Polymer Detection System according to the manufacturer’s instructions. The slides were lightly counterstained with hematoxylin and mounted. Appropriate positive and negative controls were used throughout.

Scoring was performed by consensus of two observers (PvD. and MHvV.). DDX3 shuttles between the nucleus and cytoplasm [26]. Since we previously observed distinct cytoplasmic and nuclear expression patterns, we allocated separate scores to cytoplasmic and nuclear DDX3 expression, as before [5]. Almost all cells expressed cytoplasmic DDX3, but the intensity varied and was therefore scored semi-quantitatively as absent (0), weak (1), moderate (2) or strong (3). The optimal cut-off point was selected using the online tool cut-off finder, which helps to select a cut-off that maximizes the difference in survival between groups [27]. Cases with score 0 to 2 were classified as having low DDX3 expression and evaluated against cases with high (score 3) expression, as before [6]. Cytoplasmic DDX3 was considered upregulated when DDX3 expression was low in the primary tumor (0–2) and high in the metastasis (3). Although the intensity of nuclear DDX3 in cells was similar, the fraction of positive cells varied. Therefore, the percentage of DDX3 positive nuclei was scored, regarding samples with ≥1% DDX3 staining as positive. When nuclear DDX3 was absent from the primary tumor and present in the metastasis, nuclear DDX3 was considered upregulated.

### Statistics

Dichotomized cytoplasmic and nuclear DDX3 scores in primaries and metastases of the same patient were compared. Paired odds ratios were calculated by taking the ratio of discrepant pairs. p-values were calculated by McNemar’s test. Correlations between high DDX3 in metastases and other clinicopathological variables were studied. Discrete variables were compared by χ^2^ or Fisher’s exact test. The distribution of continuous variables was assessed graphically and Student’s *t* tests or Mann Whitney U-tests were used for normally and non-normally distributed variables, respectively. Overall survival data from the time of biopsy of metastatic lesions onward was available for 58 patients and compared between patients with low verses high metastatic DDX3 expression by plotting Kaplan–Meier curves and performing modified Wilcoxon tests. Potential confounders were analyzed by including variables associated with both DDX3 expression and survival in a multivariate cox-proportional hazards model. Effect measure modification was explored by including multiplicative interaction terms in a Cox proportional-hazards model. If sample size allowed stratified analysis was performed in the case of significant interaction. P-values smaller than 0.05 were considered statistically significant. All statistical analyses were performed with R version 3.2.0.

## Results

### DDX3 is overexpressed in breast cancer metastases

**Fig. 1:**
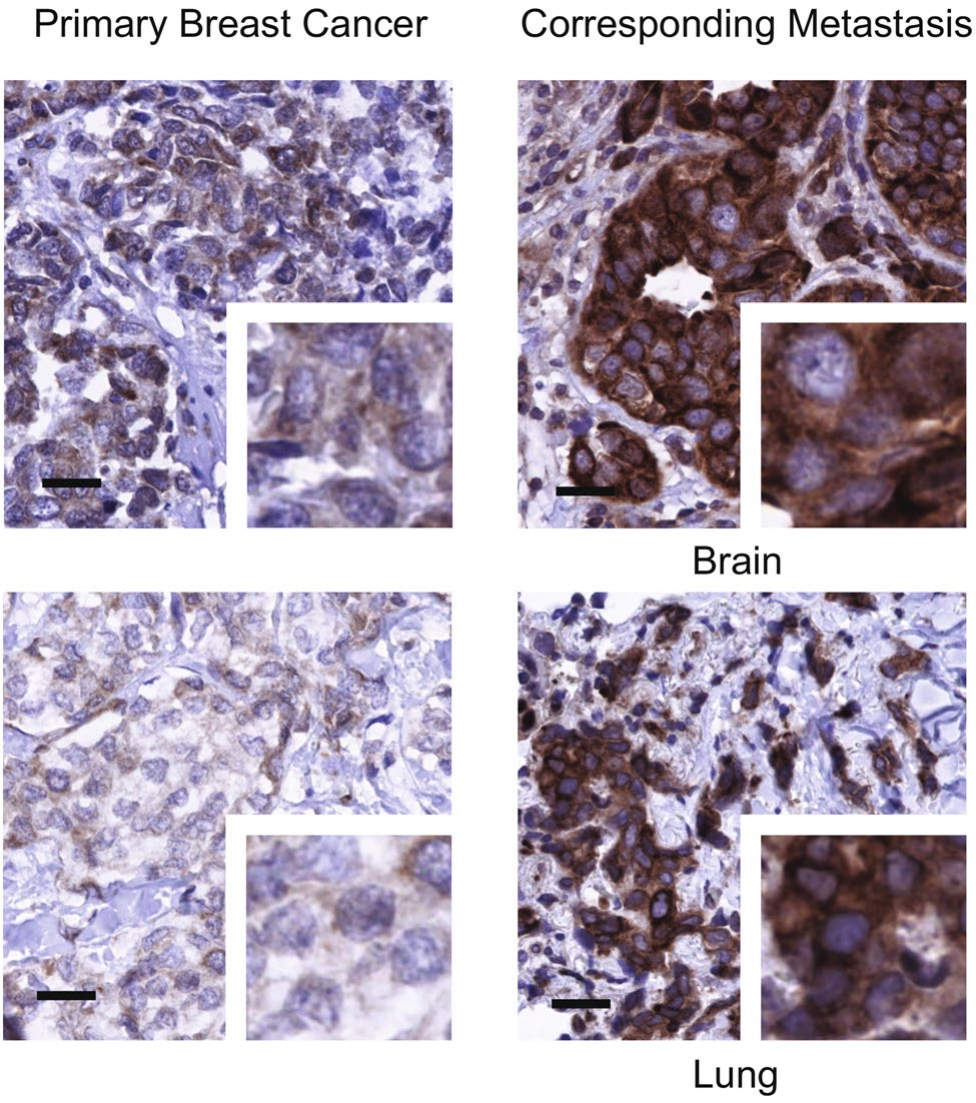
Cytoplasmic DDX3 expression is upregulated in breast cancer metastases. Examples of upregulation of cytoplasmic DDX3 expression in breast cancer metastases at different anatomical locations as compared to the originating primary breast cancer in the same patient. Analysis was performed in 79 pairs, ×40 magnification, scale bar indicates 25 μm

DDX3 could be assessed in 79 paired primary breast cancers and corresponding metastases. High cytoplasmic DDX3 expression was observed in 19% of primary breast cancers and 39% of metastases. Pairwise analysis of primary tumors and metastasis in the same patient showed that 28% of metastases had upregulated DDX3 expression, whereas DDX3 was downregulated in only 8% of patients (Table 1). This difference was highly statistically significant with an OR of 3.7 (95% CI 1.58–8.51; p = 0.002). Figure 1 shows examples of increased cytoplasmic DDX3 expression at different metastatic sites. DDX3 expression was especially prominent in breast cancer brain metastases, with 65% of metastases having high DDX3 expression and 48% of patients having an increase as compared to their primary tumor (OR 15.0, 95% CI 3.29–68.34, p < 0.001, Table 1). Upregulation of cytoplasmic DDX3 expression was also common in lung (20%) and skin (20%) metastases. The low number of available liver (n = 3) and bone (n = 3) metastases did not allow subgroup analysis for these specific anatomical sites.

**Table 1:** Changes in cytoplasmic DDX3 expression in breast cancers from primary to metastatic tumors at different sites

	N	Cytoplasmic DDX3			
		High to low	Low to high	OR (95% CI)	p-value
Total	79	6 (8%)	22 (28%)	3.7 (1.58–8.51)	0.002
Brain	31	1 (3%)	15 (48%)	15.0 (3.29–68.34)	<0.001
Lung	15	0 (0%)	3 (20%)	–	0.083
Skin	20	1 (5%)	4 (20%)	4.0 (0.53–30.31)	0.180
Other	13	4 (31%)	0 (0%)	–	0.046

Paired odds ratio (OR) is calculated by taking the ratio of discrepant pairs. Paired p-values are calculated by McNemar’s test

Nuclear DDX3 expression was observed in 22% of primary breast cancers and 13% of metastases. As shown in Table 2, conversion from nuclear DDX3 from absent in the primary tumor to present in the metastasis occurred in 9% of pairs, whereas the opposite occurred in 18% of patients (OR 0.5; 95% CI 0.21–1.22; p = 0.127).

**Table 2:** Changes in nuclear DDX3 expression in breast cancers from primary to metastatic tumors at different sites

	N	Nuclear DDX3			
		Present to absent	Absent to present	OR (95% CI)	p-value
Total	79	14 (18%)	7 (9%)	0.5 (0.21–1.22)	0.127
Brain	31	5 (16%)	3 (10%)	0.6 (0.15–2.47)	0.480
Lung	15	3 (20%)	0 (0%)	–	0.083
Skin	20	5 (25%)	2 (10%)	0.4 (0.08–1.95)	0.257
Other	13	1 (8%)	2 (15%)	2.0 (0.19–21.04)	0.564

Paired odds ratio (OR) is calculated by taking the ratio of discrepant pairs. Paired p-values are calculated by McNemar’s test

### Metastatic cytoplasmic DDX3 overexpression correlates with triple-negative receptor status and high mitotic activity

In order to catalogue what characterized patients with high metastatic DDX3, we explored correlations with other clinicopathological characteristics (Table 3). High cytoplasmic DDX3 in metastasis was associated with a higher mitotic activity index (MAI) (30.3 vs. 21.3; p = 0.033) and triple-negative molecular subtype (43% vs. 24%; p = 0.019) in the primary tumor and negative estrogen receptor (ER) status in the metastasis (72% vs. 45%; p = 0.043). Since DDX3 in metastases was associated with ER-negativity and possible negative selection pressure occurred on ER expression when patients were treated with hormonal treatment, we assessed whether adjuvant treatment of the primary tumor correlated with metastatic DDX3 expression. No correlation was found between high DDX3 in metastases and chemotherapy (47% vs. 54%; p = 0.795), hormonal therapy (21% vs 27%; p = 0.790) or treatment with trastuzamab (3% vs. 0%; p = 1). Nuclear DDX3 in metastases was associated with negative HER2 receptor status in the metastasis, but did not correlate with other clinicopathological variables (supplementary table 1).

**Table 3:** Correlation between cytoplasmic DDX3 expression and clinicopathological variables in breast cancer metastases

	Cytoplasmic DDX3			
	Total	Low	High	p-value
Characteristics primary tumor				
Tumor size in cm, median (IQR)	7 (2)	7 (2.75)	7 (2)	0.657^#^
Histology, n (%)				
Ductal	67 (86)	39 (81)	28 (90)	0.857**
Lobular	8 (10)	6 (13)	2 (6)	
Metaplastic	3 (4)	2 (4)	1 (3)	
Apocrine	1 (1)	1 (2)	0	
Grade, n (%)				
I	1 (1)	1 (2)	0	0.670**
II	21 (27)	14 (30)	7 (23)	
III	55 (71)	31 (67)	24 (77)	
Missing	2	2	0	
MAI, mean (SD)	24.8 (19.7)	21.3 (18.6)	30.3 (20.5)	0.033^$^
Lympnodes, n (%)				
Negative	39 (49)	28 (58)	11 (35)	0.080*
Positive	40 (51)	20 (42)	20 (65)	
Age, mean (SD)	52.2 (11.0)	54.0 (11.0)	49.5 (10.4)	0.074^$^
Missing	1	1	0	
Molecular subtype, n (%)				
HER2-enriched	11 (15)	5 (12)	6 (20)	0.019**
Luminal A	29 (41)	23 (56)	6 (20)	
Luminal B	8 (11)	3 (7)	5 (17)	
Triple negative	23 (32)	10 (24)	13 (43)	
Missing	8	7	1	
Characteristics metastasis				
Location, n (%)				
Brain	31 (39)	11 (23)	20 (65)	0.001*
Skin	20 (25)	15 (31)	5 (16)	
Lung	15 (19)	10 (20)	5 (16)	
Other	13 (16)	12 (25)	1 (3)	
Estrogen receptor, n (%)				
Negative	39 (57)	18 (45)	21 (72)	0.043*
Positive	30 (43)	22 (55)	8 (28)	
Missing	10	8	2	
Progesterone receptor, n (%)				
Negative	39 (57)	26 (67)	22 (81)	0.295*
Positive	30 (43)	13 (33)	5 (19)	
Missing	13	9	4	
HER2 receptor, n (%)				
Negative	48 (73)	31 (82)	20 (69)	0.363*
Positive	18 (27)	7 (18)	9 (31)	
Missing	12	10	2	
Nuclear DDX3, n (%)				
Absent	69 (87)	42 (79)	27 (26)	1**
Present	10 (13)	6 (21)	4 (74)	

P-value calculated by *Chi square test, **Fisher exact test, ^#^Mann–Whitney U test, ^$^student’s *t* test

### Metastatic DDX3 expression correlates with worse survival

**Fig. 2:**
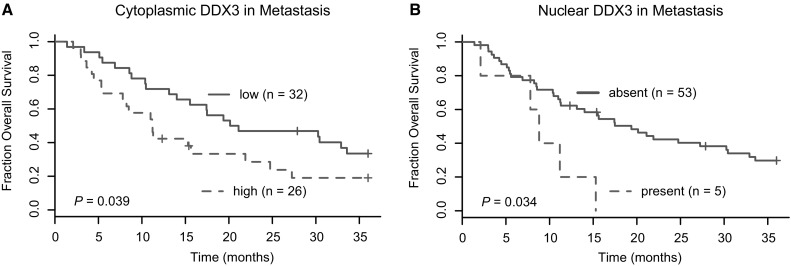
Metastatic DDX3 is associated with shorter survival in distant breast cancer metastases. **a** Kaplan–Meier curves showing overall survival after biopsy of the metastasis in breast cancer patients with high cytoplasmic (n = 26) as compared to those with low cytoplasmic (n = 32) DDX3 expression in the metastatic lesion. **b** Kaplan–Meier curves showing overall survival after biopsy of the metastasis in breast cancer patients with nuclear DDX3 (n = 53) as compared to those without nuclear DDX3 (n = 5) in the metastatic lesion. P-values calculated by a modified Wilcoxon test

We performed survival analysis to see whether DDX3 expression correlated with clinical outcome in metastatic breast cancer patients (Fig. 2). Median overall survival after the metastatic lesion was biopsied was shorter in patients with high cytoplasmic DDX3 (11.18 months) when compared to patients with low cytoplasmic DDX3 (20.14 months; HR 1.79; 95% CI 0.97–3.33; p = 0.039). Because the molecular subtype of the primary tumor and the location and ER-status of the metastasis were associated with both high cytoplasmic DDX3 and survival, potential confounding by these factors was explored in a multivariate model as much as sample size permitted. The association between cytoplasmic DDX3 and survival weakened after adjustment for individual covariates by Cox-regression analysis. This indicates that molecular subtype (HR_adjusted_ 1.38; 95% CI 0.73–2.63; p = 0.324), ER status (HR_adjusted_ 1.51; 95% CI 0.81–2.82; p = 0.200) and location of the metastasis (HR_adjusted_ 1.52; 95% CI 0.9–2.94; p = 0.210) are confounding the relation between cytoplasmic DDX3 and survival.

In addition, a significant correlation between the presence of nuclear DDX3 in metastases and overall survival was observed. Patients with nuclear DDX3 had a shorter median survival of 8.8 vs. 19.4 months (HR 3.28; 95% CI 1.23–8.75; p = 0.034). Unfortunately multivariate analysis was not possible due to the low number of patients with nuclear DDX3 in the metastasis. Overall we conclude that there is a relation between metastatic DDX3 expression and survival, which for cytoplasmic DDX3 can in part be attributed to the molecular subtype and location of these tumors.

## Discussion

DDX3 is an RNA helicase with oncogenic properties, which has been found to promote metastasis in functional studies. However, DDX3 expression had never been specifically evaluated in metastatic cancer patient samples. In this study, we therefore compared DDX3 expression in primary breast cancers to that in corresponding distant metastatic lesions. Cytoplasmic DDX3 expression was significantly higher in metastatic cancer samples, especially in brain metastases and triple negative cases. In addition, there is a correlation between DDX3 expression in the metastasis and worse overall survival in patients with metastatic breast cancer.

Previous studies have indicated that DDX3 overexpression facilitates dissemination of cancer cells through induction of an EMT phenotype [10–12]. Increased motility and anchorage independent growth have also been linked to the role DDX3 has in mRNA translation. Chen, et al. found DDX3 to increase invasive properties through a direct role in Rac1 translation, which in its turn stabilizes β-catenin expression resulting in activated Wnt-signaling [14]. Furthermore, Hagerstrand, et al. found that DDX3 mediates IRES-dependent translation, resulting in increased anchorage independent growth in cancers with 3q26 amplification. In addition to promoting the dissemination process, our finding that among patients with established metastases, those with DDX3 expression have worse overall survival is suggestive of DDX3 also providing a survival benefit to cancer cells after colonization of the metastatic niche. However, this difference can also be partly attributed to the frequent triple negative phenotype and brain localization of metastases with high cytoplasmic DDX3 expression. Notably, there are some contradictory reports in literature pointing towards DDX3 functioning as a tumor suppressor [28, 29]. It is possible that the role of DDX3 in oncogenesis differs between genetic backgrounds and cancer types [30].

The mechanisms behind cytoplasmic overexpression and nuclear retention of DDX3 remain largely to be elucidated. Mutations in DDX3 have been detected in medulloblastomas [31], head and neck cancers [32] and hematological malignancies [33, 34], but were not identified in breast cancers [35]. In addition, there is no amplification of the DDX3 locus in DDX3 overexpressing breast cancer cell lines [10]. With regard to nuclear retention of DDX3, we know that DDX3 is exported out of the nucleus as part of messenger ribonucleoprotein complexes [2, 26, 36]. In the nucleus, DDX3 was previously found to localize to the nucleolus [37] where ribosomal assembly takes place, suggesting that nuclear DDX3 retention in metastases possibly reflects increased demand in protein synthesis. More research to further clarify the mechanisms of DDX3 overexpression and nuclear retention is needed.

We found a particularly large increase in cytoplasmic DDX3 expression rates in brain metastases. Brain metastases are more common in patients with triple negative or HER2 amplified primary breast cancers [38], which have relatively high DDX3 expression. However, discordance rates for DDX3 were much higher (48% upregulation) than for HER2 (2%) and estrogen receptor (13%) [21]. It is therefore hard to explain the DDX3 upregulation in brain metastases rates solely by an association with these molecular subtypes. Several other biological signatures have been found to characterize brain metastases. Wnt signaling mediates metastasis to the brain in both lung [39] and breast cancer [40]. DDX3 is a multilevel activator of the Wnt-signaling pathway [5, 6, 14, 41] and therefore potentially facilitates brain colonization in a Wnt-mediated fashion. Another feature of brain metastases is the expression of DNA repair genes [42, 43]. Inhibition of DDX3 reduced non-homologous end joining, a double strand break repair mechanism [5], implying that the high DDX3 levels in brain metastases could reflect a DNA damage response. Furthermore, overexpression of hypoxia-inducible factor 1α is common in brain metastases [44] and also associated with DDX3 expression in breast cancer [45]. However, metastatic DDX3 expression did not correlate with expression of the HIF-1α target genes carbonic anhydrase IX (CAIX) and Glucose transporter 1 (GLUT-1; data not shown), making it unlikely that high DDX3 expression in brain metastases is hypoxia-mediated. Last, metastatic niches differ also by the bioenergetic profile they impose on cells [46]. Brain metastases were demonstrated to upregulate glycolysis and oxidative phosphorylation capacity [47] and to have increased hexokinase 2 expression [48]. An additional reason for brain metastases to elevate DDX3 expression could be that DDX3 supports metabolic adaptation of cancer cells to the microenvironment of the brain. Although liver and bone metastases are also common in breast cancer patients, limited availability of tissue from these sites did not allow for subgroup analysis.

Besides biological relevance, high DDX3 expression in breast cancer metastases has potential clinical applications. Metastatic breast cancer, especially localized in the brain, is associated with short patient survival and severely impaired quality of life. Cerebral metastases occur early in triple negative cases [49], where the systemic therapeutic arsenal is particularly lacking. High DDX3 expression could serve as a therapeutic target in these patients. There are several small molecule inhibitors of DDX3 currently under development [50]. Although diffusion of these compounds over an intact blood brain barrier is limited [5], the small size of the inhibitors and the compromised blood brain barrier in brain metastases [51] potentially do allow for therapeutic levels to be reached. The DDX3 inhibitor RK-33 has potent radiosensitizing abilities [10], which could enhance the effect of whole brain radiation to treat brain metastases. Furthermore, given the role of DDX3 early in the metastatic process, DDX3 inhibitors could potentially also be used to prevent the emergence of metastases. At last, evaluation of DDX3 expression in patient samples could serve both as a prognostic biomarker and facilitate selection of those patients benefiting most from DDX3 inhibitors.

## Conclusions

Cytoplasmic DDX3 expression is increased in breast cancer metastases, especially those located in the brain and occurring in triple negative cases. In addition, patients with high DDX3 levels in the metastatic lesion have shorter overall survival, implying that DDX3 is a potential therapeutic target in metastatic breast cancer.

## Electronic supplementary material

Below is the link to the electronic supplementary material.

Supplementary material 1 Correlation between nuclear DDX3 expression and clinicopathological variables in breast cancer metastases (DOCX 15 KB)

## Abbreviations

DDX3: DEAD box RNA helicase 3
MAI: Mitotic activity index
ER: Estrogen receptor
PR: Progesterone receptor
HER2: Human epidermal growth factor receptor
OR: Odds ratio
95% CI: 95% confidence interval
HR: Hazard ratio
TN: Triple negative
EMT: Epithelial to mesenchymal
TMA: Tissue microarray
