# Supplementary material for: The prognostic effect of DDX3 upregulation in distant breast cancer metastases
Source: Clin Exp Metastasis. 2016 Dec 20;34(1):85–92. doi: 10.1007/s10585-016-9832-8 (PMC5285427; doi:10.1007/s10585-016-9832-8)
Supplement: Supplementary file 1 — Supplementary material 1 Correlation between nuclear DDX3 expression and clinicopathological variables in breast cancer metastases (DOCX 15 KB) [file 10585_2016_9832_MOESM1_ESM.docx]

Supplementary table 1 Correlation between nuclear DDX3 expression and clinicopathological variables in breast cancer metastases.

|  |  | **Nuclear DDX3** | |  |
| --- | --- | --- | --- | --- |
|  | **Total** | **Absent** | **Present** | **P-value** |
| **Characteristics primary tumor** |  |  |  |  |
| **Tumor size in cm, median (IQR)** | 7 (2) | 7 (2) | 7 (1.75) | 0.868# |
| **Histology, n (%)** |  |  |  |  |
| **Ductal** | 67 (85) | 57 (83) | 10 (100) | 0.763** |
| **Lobular** | 8 (10) | 8 (12) | 0 |  |
| **Metaplastic** | 3 (4) | 3 (4) | 0 |  |
| **Apocrine** | 1 (1) | 1 (1) | 0 |  |
| **Grade, n (%)** |  |  |  |  |
| **I** | 1 (1) | 1 (1) | 0 | 0.754** |
| **II** | 21 (27) | 19 (28) | 2 (20) |  |
| **III** | 55 (70) | 47 (70) | 8 (80) |  |
| **missing** | 2 | 2 | 0 |  |
| **MAI, mean (SD)** | 24.8 (19.7) | 24.7 (20.0) | 25.5 (18.8) | 0.973$ |
| **Lympnodes, n (%)** |  |  |  |  |
| **negative** | 39 (49) | 35 (51) | 4 (40) | 0.737** |
| **positive** | 40 (51) | 34 (49) | 6 (60) |  |
| **Age, mean (SD)** | 52.2 (11.0) | 52.3 (10.9) | 52.0 (11.8) | 0.951$ |
| **missing** | 1 | 1 | 0 |  |
| **Molecular subtype, n (%)** |  |  |  |  |
| **HER2-enriched** | 11 (15) | 7 (11) | 4 (44) | 0.070** |
| **luminal A** | 29 (41) | 26 (42) | 3 (33) |  |
| **luminal B** | 8 (11) | 7 (11) | 1 (11) |  |
| **triple negative** | 23 (32) | 22 (35) | 1 (11) |  |
| **missing** | 8 | 7 | 1 |  |
| **Characteristics metastasis** |  |  |  |  |
| **Location, n (%)** |  |  |  |  |
| **brain** | 31 (39) | 27 (39) | 4 (40) | 1** |
| **skin** | 20 (25) | 18 (26) | 2 (20) |  |
| **lung** | 15 (19) | 13 (19) | 2 (20) |  |
| **other** | 13 (16) | 11 (16) | 2 (20) |  |
| **Estrogen receptor, n (%)** |  |  |  |  |
| **negative** | 39 (57) | 34 (56) | 5 (63) | 1** |
| **positive** | 30 (43) | 27 (44) | 3 (38) |  |
| **missing** | 10 | 8 | 2 |  |
| **Progesterone receptor, n (%)** |  |  |  |  |
| **negative** | 39 (57) | 43 (74) | 5 (63) | 0.673** |
| **positive** | 30 (43) | 15 (26) | 3 (38) |  |
| **missing** | 13 | 11 | 2 |  |
| **HER2 receptor, n (%)** |  |  |  |  |
| **negative** | 48 (73) | 47 (81) | 4 (44) | 0.030** |
| **positive** | 18 (27) | 11 (19) | 5 (55) |  |
| **missing** | 12 | 11 | 1 |  |

P-value calculated by * chi-square test, ** Fisher exact test, # Mann-Whitney U test, $ student’s t-test.
